# Communication in healthcare: experience of students and professionals from teaching- learning to practice in health

**DOI:** 10.5116/ijme.6412.f49b

**Published:** 2023-03-30

**Authors:** Fernanda Patrícia S. S. Novaes, João G.B. Alves, Suely Grosseman

**Affiliations:** 1 Department of Medicine, Universidade Federal do Vale do São Francisco (Univasf), Brazil; 2 Postgraduate Program, Instituto de Medicina Integral Professor Fernando Figueira (IMIP), Brazil; 3 Postgraduate Program in Medical Sciences, Federal University of Santa Catarina (UFSC), Brazil

**Keywords:** Healthcare communication, medical education, teaching-learning, focus groups, philosophical hermeneutics

## Abstract

**Objectives:**

This study aimed to understand the teaching-learning experience in the Communication in Healthcare class among students, teaching assistants, and health professionals, as well as its applications to professional practice.

**Methods:**

This is a qualitative study with a theoretical approach based on Gadamer’s Philosophical Hermeneutics and a methodological framework based on Minayo and Bardin’s thematic content analysis. Communication in Healthcare is an elective multiprofessional class, which lasts one semester and is offered regularly. All former students (n = 368) were invited to participate by email, and 30 participated in these focus groups (13 students, 8 teaching assistants, and 9 health professionals). The online focus groups took place on an online platform, and they were video-recorded and subsequently transcribed. Through cross-sectional and vertical analysis, the main themes were identified.

**Results:**

The Communication in Healthcare class was an important step for personal, professional, and interprofessional formation and development of communication competence. The following dominant themes were identified: 1) motivation for signing up, 2) prior expectations, 3) meaning of the experience and shaping moments, 4) how the teaching-learning experience was retained and what was retained, 5) repercussions in relation to self, others, and professional life, and 6) reflections about the curriculum, interprofessional dialogue, and formation.

**Conclusions:**

The teaching-learning experience was important for the formation of communicational competence. This research contributes to medical education and opens teaching-learning paths for communication skills, empathy, dialogue, and interprofessionalism. Future studies with a philosophical hermeneutic framework and online focus groups are indicated for the comprehension of educational interventions in health.

## Introduction

Communication and interprofessional teamwork are fundamental to optimal outcomes in healthcare, as they are the basis of good professional practice.^1^^,^^2^ Communication training has been included in the curricula of health professions due to the realization that students lacked preparation to deal with their own subjectivity and that of others; due to health professionals’ limited ability to listen attentively to patients and people involved in the care process, dialogue with them, and address psychosocial, cultural, and spiritual dimensions; and due to negative outcomes arising from the lack of health communication.^1^^,^^3^^,^^4^ 

The essential nature of health communication and the realization that it is possible to teach it have led diverse countries to develop recommendations and consensuses on what aspects of communication should be taught, as well as how they should be taught, in medical schools and other areas of health.^5^^-^^11^ Thus, several well-established strategies have been applied for effective communication teaching, such as role-playing, dramatization and the platform DocCom.^12^^,^^13^

In Brazil, since 2001, guidelines for undergraduate courses in the health area have included communication as one of the competencies expected of future health professionals. However, not all universities have systematically included communication in their curricula.^14^^-^^16^ In addition to the challenges that administrators and educators face in agreeing upon including it in curricula, there is the challenge of preparing educators to teach communication by means of teacher development.^17^

In 2015, at a public Brazilian university, located in the region of the São Francisco Valley, an optional/elective, multiprofessional class called “Communication in Healthcare” (CH) was created to advance communication teaching, and it has been offered to students of Medicine, Nursing, Pharmacy, and Psychology. CH combines several strategies, some of which are innovative. Since its implementation, student demand has increased. The first semester the class was offered, 4 students signed up, and this number gradually increased to 80 students per semester. By the year 2020, more than 400 students had participated in CH.^18^ Motivated by their experience in the class, many students went on to voluntarily become teaching assistants (TAs) during subsequent semesters.

Qualitative studies have investigated the perspectives of students, residents, and health professionals on communication skills training.^19^^-^^21^

However, there is a knowledge gap regarding the experience of students, TAs, and health professionals who have taken optional/elective, multiprofessional classes during their course of studies, providing evidence of that they think today about the past educational experience.

With the aim of contributing to this knowledge gap, the objective of this study was to understand the teaching-learning experience in CH among students, TAs, and health professionals who took the class, as well as how they applied it in their professional practice.

## Methods

Study design and participants

This is a qualitative study with cross-sectional and vertical thematic content analysis. The theoretical framework is based on Gadamer’s Philosophical Hermeneutics,^22^^-^^26^ and the methodological framework is based on Bardin’s and Minayo’s approach.^27^^,^^28^ Approaches that articulate the fields of Philosophy and Medicine undergo transformations whose conception is fundamentally important to a deeper understanding of interpersonal interaction, life, and death with respect to people who receive care.^29^ Philosophical hermeneutics is a comprehensive approach that brings the researcher closer to the research object. It is suitable for studying relational research objects, such as research in education that involves the context and inter-subjective experience of the student-teacher relationship, in addition to contexts in the health area, which involve care and the doctor-patient relationship, for example, a study that applied content analysis informed by Philosophical Hermeneutics to understand the thinking and acting of health professionals at the level of care.^30^

Eligible participants were the 368 students who had taken the class between the first semester of 2015 and the second semester of 2019. They were all invited to participate in the study by means of an email that stated the study’s objectives and ethical guidelines, guaranteeing confidentiality and anonymity. Of those invited, 40 accepted the invitation.

The sampling procedure was purposeful for the pilot focus group (FG) and by invitation for the other FGs, with the aim of maintaining spontaneity in participation in the study, similarly to CH, where students sign up of their own volition.

In this context, the sampling procedure considered a limit of FG participants (approximately 8 to 12 per group), according to the order in which they responded to the invitation to participate. Participants were distributed into 3 FGs, divided as follows: students, TAs, and health professionals. This distribution aimed for data saturation.

There was an additional pilot FG, with purposeful sampling of 4 coauthors of an article, to test their understanding of the guiding questions, the socio-demographic information form, access to the virtual platform, and video-recording of the online meeting.

The FGs were divided into the following groups: 1. Students: students who had taken the class and had not yet graduated; 2. Teaching assistants (TAs): students who, in addition to taking the class, went on to become TAs; and 3. Health professionals: former students who had already graduated. Six participants were unable to participate due to technical difficulties, resulting in a total of 30 participants.

During the interview, the participants filled out a form with the following socio-demographic characteristics: sex, age, area of health, year in university or whether graduated, and type of participation in the class. The socio-demographic characteristics are displayed in Table 1.

Participants were between the ages of 20 and 47 years. In the Results of this manuscript, the interviews were identified according to the following information: interviewee’s FG, gender, and area of study.

The research project received ethical approval from the Institutional Ethics Committee for Research Involving Human Beings under opinion number 3.997.525 and CAAE 30070620.4.0000.5201. The study followed Resolutions 466/2012 and 510/2016 of the Brazilian National Research Ethics Commission, which provide guidelines and regulatory standards for research involving human beings, and the study was carried out in accordance with the Declaration of Helsinki. Before initiating information collection, all participants read and signed the free and informed consent form.

**Table 1:** Socio-demographic characteristics of the focus groups

Characteristics	Teaching assistants FG-TA	Students FG-S	Health professionals FG-HP
Number of participants	8	13	9
Female sex	4	7	4
Male sex	4	6	5
Area of Health: Medicine (Med), Nursing (Nur), Pharmacy (Pha), and Psychology (Psy)	Med, Nur, Pha, and Psy	Med, Nur, Pha, and Psy	Med and Nur

### The educational intervention

The education intervention was CH, an elective multiprofessional class that was developed by the first author, with diverse strategies that together formed an innovative path, including creative suggestions from students, in an ongoing process of dialogue and feedback.^31^^,^^32^ CH lasts 6 months, with weekly 4-hour meetings, receiving approximately 40 to 80 students who sign up of their own accord.

The teaching-learning and assessment strategies involve: 1. The platform DocCom.Brasil; 2. Ludic-artistic-reflective seminars; 3. Assessment via adapted, collaborative Objective Structured Clinical Examination (OSCE); 4. Interprofessional learning, 5. Activities to promote well-being in the academic environment.

The platform DocCom.Brasil is a didactic tool for teaching medical communication developed in partnership by the American Association on Communication in Healthcare and Drexel University College of Medicine,^33^ with 12 of the original 40 modules translated to Portuguese by the third author of this study, in partnership with Fernanda Udinal, who is a technical translator at the Blood Center of the University of Ribeirão Preto, São Paulo, Brazil; some of the translated modules have also been validated by scientific studies.^34^

The ludic-artistic-reflective seminars intentionally work with cognitive, attitudinal, and procedural domains of health communication, applying ten steps: 1. Class project prepared by the facilitating group under supervision of the professor and TAs; 2. Division of the class group into subgroups for reflection; 3. Conversation circle with reflective discussion about DocCom.Brasil questions; 4. Categorization of responses into themes; 5. Search for articles related to the themes and sharing as a group; 6. Integrative dynamics (student-led games for interaction and learning about the theme of the meeting); 7. Expository class with dialogue on the theme of the DocCom.Brasil module; 8. Videos produced by students reflecting communication attitudes; 9. Dramatization; 10. Conclusion with art.^35^ Student productions bring audiovisual material, poetry, music, games, and integrative dynamics that make CH a laboratory of sensibilities, where each meeting is an unprecedented experience.

The OSCE has been adapted to occur in collaboration with students and TAs, who assume the role of co-authors and actors in OSCE stations and develop their own experience in these roles.^36^

Strategies for promoting care and well-being in academic life include the following: the “angel dynamic” where each student is randomly and secretly assigned a person to take care of by means of motivational messages, small gifts, or anything else that stimulates happiness;^37^ the teaching-learning setting of a non-conventional classroom (known as the “blue room”) with pillows and mats that facilitate dynamics and role-playing to contextualize clinical situations;^38^ group hugs at the end of each meeting in a large circle, where each student says a word to summarize their experience during that class meeting.^39^ The class promotes interprofessional learning by developing collaborative competencies for teamwork.^40^

### Setting

The medical school is part of a public federal university located in the interior of the Northeast Region of Brazil, in the Caatinga Biome, which is a hot, semi-arid region with very challenging conditions. This medical school, which lasts 6 years, does not have any mandatory classes on communication, and CH is the only formal approach to teaching communication.

CH, an elective multiprofessional class that lasts 6 months, is an educational intervention that introduces active methods and teaching-learning communication into a traditional Brazilian medical school.

Following ethical approval, information was collected in 2020, by means of FGs, using an online platform. Each FG lasted from 90 to 110 minutes. In-person FGs, as planned in the initial project, were substituted by online FGs, in following with the historical-epidemiological moment of the COVID-19 pandemic, meeting the need for social distancing and guaranteeing research participants’ biosafety.^41^ The main reason for choosing FGs was to gather detailed information on the theme from a group of participants.^42^^,^^43^

In order to comprehend the participants’ experience, the following 3 FGs were formed: one with 13 former students (FG-S); one with 8 former students who had also gone on to become TAs (FG-TA); and one with 9 health professionals who had taken the class when they were students (FG-HP).

The first author observed and participated in all FGs (FG-S, FG-TA, and FG-HP). A psychologist conducted the interviews in FG-S and FG-TA, and the third author conducted the interview in FG-HP. The first author developed the educational intervention, and she had taught the course when the participants attended. The third author participated in the project that translated the online platform Doctor Communication to Brazilian Portuguese to create DocCom.Brasil.

The following guiding questions were developed by the first author:

How was the experience in the CH class, and what were the repercussions for the construction of the participants’ “being”? (defining moments/memories/experiences):

§  Regarding acquisition of health communication abilities?

§  Regarding teaching-learning mediated by art in the ludic-artistic-reflective seminars?

§  Regarding the platform DocCom.Brasil?

§  Regarding assessment via OSCE?

§  Regarding contact with other students of health during class meetings?

§  Regarding the setting of the blue room?

After the participants filled out the socio-demographic form, all FG interviews were video-recorded using the online platform. A research assistant subsequently transcribed all interviews verbatim, and the transcriptions were sent to all participants for their evaluation and approval. All participants approved the transcriptions without any suggestions. Another research assistant, who is a native speaker of English, translated all quotations from Brazilian Portuguese.

### Data analysis

Thematic content analysis was performed, from a hermeneutic approach, seeking to fuse horizons of perspective dialectically.^22^^-^^25^^,^^44^ The steps of analysis were: floating reading (pre-analysis) of the FG transcripts, which consists of initial reading to acquire familiarity with what was expressed. Subsequently, the text was annotated with colored pens and notes in the margins. This was followed by grouping which formed the basis of themes in a process of cross-sectional and vertical analysis of speech, an action that recalls the attitude of comprehension, empathy, and immersion in the subjective world of the other.^27^^,^^45^ Data saturation was reached with the number of FG interviews, making in unnecessary to conduct further interviews.

## Results

One of the main findings from the focus groups was that the teaching-learning experience represented an important step for personal, professional, and interprofessional formation and development of communication competence.

### Vertical analysis

Beginning with FG-TA, a thematic category that was unique to this group was the motivation for being a TA. In this group, art was cited as a motivation for taking the class as a student. The themes of autonomy to make decisions and the challenge of conducting the class and the OSCE using the available resources were also emphasized. This FG brought reflections comparing academic training to a “steamroller” and reflections about the positive meaning of CH in students’ mental health, thus breaking paradigms.

FG-S was the only one that spoke about expectations in relation to the CH class, and one of the participants reported that they had imagined that the class would follow a more theoretical pattern, like other classes in the course, but the student was surprised by the practices of dramatization, strategies with art, and others. This FG emphasized the student-teacher relationship and art as defining aspects, in addition to citing other steps of the teaching-learning method.

FG-HP emphasized learning and repercussions of the experience on professional life. They discussed communicating a child’s death to family members, as well as the meaning of the experience in CH as an important basis for not giving up on Medicine at a time like this. They also highlighted the importance of team communication and multidisciplinary work in cases of gender-based violence and learning that was retained about bad news, sexuality, and other topics. As in other groups, statements about the presence of the class in daily practice were recurrent. They underscored that all students need to live these moments during the health education process, because they need to learn assertive and sensitive treatment with patients. They also emphasized that CH brings warmth to interpersonal relationships.

### Cross-sectional analysis

The dominant themes of cross-sectional and vertical comprised: 1) motivation for signing up, 2) prior expectations, 3) meaning of the experience and shaping moments, 4) how the teaching-learning experience was retained and what was retained, 5) repercussions in relation to self, others, and professional life, and 6) reflections about the curriculum, interprofessional dialogue, and formation.

### Motivation for signing up

Among all participants, motivation for signing up for CH included the need to take an elective class, interest in the topic of communication, encouragement from friends who had taken the class, curiosity, and lectures offered by the professor:

“And one of the reasons that I wanted to take the class was also because of encouragement from colleagues who had taken it in the past, like [colleague], you know, he had spoken very highly of the class, said it had been a wonderful experience, and that really encouraged me to take it.” (FG-HP, M, Med)

The need to take a class on communication in order to become a good doctor, build good bonds, and relate to patients or people appropriately, and even the name of the course “Communication in Healthcare” in itself were also mentioned and associated with these aspects, for instance:

“… the idea of communication in health brought me a concept that I had, like , my family, my friends, people from outside, practically everyone I know outside the health area, outside the technical area and when they talked about a good health professional, of a good doctor, sometimes they were not praising this individual’s technical capability and performance, they were talking, they were praising the ability to communicate, to build, and build a bond…” (FG-S, M, Med)

Of those who became TAs after taking the class, motivations for doing so included the importance of the course; moments lived as a student, where they felt there was freedom of expression and students “could show their faces”; the feeling of belonging, where students were heard and felt they were “an integral part of the class”. They considered the experience as students rewarding, and they wanted an opportunity to learn about leadership and contribute to improving the class:

“…when I became a teaching assistant, I had the challenge of doing something different, for example, I wanted to shorten the period of the last test, which was the OSCE. I had the challenge of doing something that could improve interaction and help the students understand the issue of being collaborative and that, all together, united, they could improve the class more and more…” (FG-TA, M, Med)

The importance of the class in relation to valuing the other was also mentioned:

“I participated in the class at two different moments, right, as I was saying, in the second semester of college when everything was very new, so I had the view of a student, which also helped me to take the subjects that would come from the course, right. Trying to have this more humanized view of situations that have come up, however, in a more technical way, but bringing this more human side, that is something that the class talks about a lot, and, then, I returned to the class during my seventh semester, which is another phase of the course of studies, and it was very interesting for me, as a teaching assistant, because I had the chance to put the knowledge I had back then into practice with the groups that we assisted…” (FG-TA, F, Med).

### Prior expectations   

Participants’ expectations of the class included that of improving communication and rapport-building skills, learning to touch others with words, learning to welcome patients, and having contact with the universe of art. For some students, expectations were broken, representing a positive surprise, given that they were expecting the same pattern of other classes in the course:

“For me, this class actually has two meanings: […] Before I thought that the class would be something more theoretical and that we wouldn’t have so much practice, so it would be something more of the same, let’s put it that way. I signed up thinking it would be that way, and I was surprised in a very positive way, because just meeting colleagues from other classes at the time, […] I really liked the way it was carried out and the way the content was approached, because we saw much more than medicine, how to touch each other, we saw how to reach the other, through words, through transitional phrases.” (FG-S, M, Med )

### Meaning of the experience and shaping moments

The meanings of the experience were diverse, and some were multiple for the same student. It was appreciated as unique and rare within the university:

“…it’s something really unique […]. Not every public university has this kind of approach focused on the health area, so it’s something very rare.” (FG-S, M, Med)

In addition to these meanings, participants expressed that CH represented a seed of hope for better interpersonal relationships and respect among professionals from different health areas, which represented an experience of interdisciplinarity and empathy:

“I see the experience in the class as a little seed of hope for me… hope that the relationships between different professionals will be good.” (FG-S, F, Psy)

“I remembered two extremely important things about the class, interdisciplinarity, […] in communication and health, we learned to work with all the other areas of study that were there […], there were students from all areas, from Psychology, Medicine, Nursing, and another word that impressed me a lot […]: empathy, so I learned that empathy has an extremely important meaning, we put ourselves in the other’s shoes, we treat that person the way we would like to be treated…” (FG-S, F, Nur)

Other meanings included a safe haven, recharging batteries, confidence, overcoming prejudiced notions, and a different working dynamic with a new perspective “to take to other classes that are part of the course”. It also meant teamwork, learning from others, sharing tasks, collaboration, and a new form of responsibility, possibility of creation, horizontal relationships, personal and professional enhancement, autonomy, and caring without automatism. They also emphasized that the experience represented understanding human beings in a complex form, a differentiated perspective on other people, sensitivity, further training to communicate with others, preparation to deal with death, taking care of health, and encounter.

The student-teacher-class relationship symbolized a strengthening of the bonds between students and the teacher, between students and the class, between health students themselves, and between professionals and patients.

Shaping moments and defining memories were diverse, encompassing environmental awareness, care for students, volunteer activity, laughter, poetry recitals, musical presentations, among others. Participants stated that there is a set of values, known as the hidden curriculum, which is passed on between the lines by positive or negative attitudes. A positive example cited was the teacher arriving on a bicycle to teach classes, which speaks volumes more than words regarding environmental awareness and self-care:

“I would choose the image of the teacher arriving by bicycle to teach… I think it represents her dedication a lot, that she is very spontaneous, natural, likes nature, so this shows, the fact that she uses a bicycle, instead of a car or a motorcycle, shows how she takes care of the environment, and of her own health.”  (FG-S, M, Med)

The following is an example of a memory related to musical presentations:

“I think what was most defining for me in the class were those creative contributions by my colleagues. I remember a guy from Medicine who played a song on the guitar, and then the lights were turned off, he was in the center with the guitar, and so, he had flashing lights, like, around him and he sang a song that was very emotional.” (FG-S, F, Nur)

 The defining moments mentioned in multiple FGs included the care corridor, which was also cited as the hug corridor, whose meaning was related to welcoming people:

“One thing that was very defining for me in the class, as many of you have already said, is the issue of learning to welcome, empathy… There was a corridor, the dynamic was a hug corridor, and I was not very well one day and I was a little uncomfortable having this moment of contact with people, and then the corridor was formed. And you had to go through blindfolded and people would give you a hug. It was such a difficult day, when we live in a city far from our family and such, anyway, and then I felt everybody’s hug, and I burst into tears, in the room, and everyone felt a bit tense, like. But, so, even a class that’s there to talk about communication can be a form of welcoming, can be a way to improve your day. And then we perceive that, in the smallest things, we can improve somebody’s life, however it is, especially in the health area, because we are dealing a lot with people’s pain. Nobody goes to the hospital, to the pharmacy, to the health clinic because everything is alright, so we need to think about that a little when we treat, when we care for other people, about living together and dialoguing with other professionals as well. Because everyone has their burdens, their hard days, they have their problems, but we can’t let that reflect on who we are and especially how we act.” (FG-S, F, Pha)

### How the teaching-learning experience was retained and what was retained

In relation to how the teaching-learning experience was retained and what was retained, participants reported that the teaching-learning method contributed to the way in which the experience was retained, making it lasting. The class’s polyvalent character developed diverse skills in a single class, using theater, cinema, and scripts, also representing a form of therapy. Furthermore, a collective construction by the teacher and students underpins the class itself, and this is in harmony with the proposal of teaching communication and building knowledge, skills, and attitudes:

“For me one of the most defining aspects was the OSCE itself and also the staging that we did for the video. Because, like, we made a video of the woman. My seminar was on breaking bad news. So, we made a video showing how the ideal way and the inappropriate way would. And, so, I have the step by step of the video to this day. I was even an actress. I remember everything alright, to this day, and that’s what we apply.” (FG-HP, F, Nur)

Participants also stated that CH was fundamental to internships, professional practice, and everyday situations. CH also promoted learning about “the type of human beings we want to be in the world”, bringing elements to “make someone’s life better” and to deal with people’s pain:

“Sometimes, we are faced with everyday situations, not only in our professional environment, but they make us reflect on what type of human being, what kind of professional we want to be in the world, and this kind of reflection was really great for me, not just during the class, but also during the conversations themselves, not only the modules, the topics, and such, in the conversation circles themselves, in the ludic activities that were the conclusion, in the hug and everything. So, this leads us to really transform ourselves as human beings, not just as professionals, like [colleague] said, we need to think about what type of world we want to have, right, and if the change starts with us, we have to stop to reflect on how we are acting as human beings to also act as health professionals, and I have a very good memory of the class.” (FG, F, Pha)

It was also reported that CH promoted more preparation and safety in contact with patients. Participants stated that the class was the only source about breaking bad news in the curriculum and that it valued diverse learning strategies, parody, class, video, poems, and dynamics, highlighting that it contributed to improved teaching-learning:

“There was parody, there was class, there were videos, there were poems, there was the angel dynamic, the class deserves congratulations. I believe that if we used a little bit of this methodology in all classes, it would be better…” (FG-S, F, Psy)

All FGs referred to the importance of reflection through art during, as a mediator of the teaching-learning experience:

“And it’s also because of this art that the automatism is broken, right, which is what happens in health and care environments, right, that we are so submerged there in the routine that we don’t notice anymore, we don’t notice the other. I think the very use of art is there to awaken this, right, to break the routine, for us to see better.” (FG-S, F, Psy)

“… art, right, for us to express ourselves, for us to create, and that was very defining, so, as our colleague said, maybe we don’t remember the platform, but we certainly remember the teacher reciting poetry or us creating dramatizations and

presentations, in short, which end up being much more meaningful and continue offering learning as well.” (FG-S, F, Psy)

Regarding dialogue, the openness to freedom of expression was highlighted: 

“It wasn’t one of those conversations where you felt pressured to talk; it was one where you felt well, on account of the environment and the people.” (FG-S, F, Nur)

In relation to empathy, multiple participants highlighted the experience they had in contact with this theme during CH. They highlighted that putting themselves in the other’s place is a benefit of this subject:

“Not that the things we say are repeated, but the sensations are very, not similar, because each person is unique, but the issue of bad news, communicating bad news, empathy, putting yourself in the other’s shoes is a benefit of this class.” (FG-TA, M, Med)

Testimonies highlighted how empathy was developed and retained and how professional competence was formed, for example, the creation of a video simulating positive and negative professional attitudes in health care. This dynamic represented how the teaching-learning experience about empathy was retained:

“We made a mini-documentary, and our topic was strong emotions. And I remember that it is a topic that we carried not only for this professional environment, but for our entire lives. Because in our mini-documentary we put a nurse […] and we simulated a clinic and it was going to be a prenatal, I don’t remember exactly. But then the nurse arrived very late, she had several problems in her family and she arrived really stressed out, unfriendly, lots of stress. And then she entered the office and treated the patient really badly. And then, we did it the other way, right […] she came in and explained to the patient that she wasn’t doing so well, because she was going through some things. And I remember that it was very defining, because I imagine in our life, like, the other person who was waiting, she had no idea what was happening in the nurse’s life. […] So, it’s a principle that we take to our daily lives […] and realize the meaning of empathy in practice […], that, in our day to day routine, no matter how stressed you are, it is very difficult to control yourself? It is! And that’s where the learning is […] So, that’s a defining memory that I remember now…” (FG-S, F, Nur)

Also in relation to communication between professionals, they said that their experience in the class led to recognition of the importance of others in all areas of knowledge and emphasized respect for all team members as part of the knowledge acquired:

“… be it health, teaching, or any area, it’s a matter of teams. Knowing how to work in different areas and knowing the importance of each one, that there is no profession that is more than another, no professional is greater than another, no professional has more wisdom than another, I think that they all complement each other, and this class was there for this, to complement these professionals, so they will know how to communicate.” (FG-S, F, Psy)

The dynamics that brought dialogue in conversation circles, exchanging stories between students in CH who were studying different areas of health, was cited as an important way to retain the competence of working in an interprofessional group.

It was affirmed that, in this study, as in the class meetings, the authenticity of the participants’ speeches was honored during the FG meetings. In this manner, the student-teacher relationship and the experience in the class configure a terrain of safety and openness to dialogue, which contributed to open and authentic dialogue in the interview, in harmony with the teaching-learning proposal:

“… because when you start working together with all the members of a health team, you have respect, a better way of treating each member, you don’t become, don’t let yourself become arrogant, to the point of thinking that you alone are the author of the actions and you alone are the collaborator, so I thought this was very important for the class […] We always sat in circles, and we were talking about what we thought of this interaction, this articulation with other professionals, what we thought of this experience, the experience we had and for me it was very important, because it really broadened the spectrum of working in a team.” (FG-S, M, Med)

Significant learning was mentioned, promoted by several aspects that make the class positive, affective, and defining to professional identity. Several participants stated that it was a subject that made a “strong impression”. These aspects included ludicity and reflections that made the experience mobilizing, allowing students to leave the class meeting feeling touched. As this process is “constantly reflected upon, this communication, it is constantly reevaluated”. Participants remembered the teaching-learning experience, even after time had passed. They also commented on the dynamic aspects of CH, which helped them retain the knowledge and skills:

“It was a class that I spent an entire semester without taking a notebook, I don’t have any written notes of the days of the class meetings, and I have all the classes recorded by memory, I know everything that happened, I remember everything, because that’s how it was, everything was so dynamic and at the same time practical, so we were doing it there, debating, discussing, there was the issue of practice, the seminars, the OSCE itself, which was the evaluation, like, so, it was a very nice way for us to learn, then, it stuck well, like, it was very important.” (FG-HP, F, Nur)

Furthermo re, other aspects were underscored, including active participation of students, with the right to voice, a safe speech environment that was conducive to exercise, lived moments, and feelings, with an emphasis on people, such as putting oneself in others’ shoes, knowing how to break bad news, understanding others, and practicing lessons from the DocCom.Brasil platform, in empathy and creativity that make the experience surprising at every meeting:

“I took the course, the class was essentially made up of medical students and then, I believe, there was only one student who was a pharmacy student. And then when I returned, while monitoring, the class was made up of the most varied courses in the health area and as a discipline allows everyone to express themselves, everyone says how they feel in an environment where everyone can feel free to say what they want. thinks, to tell his experiences. I was able to perceive experiences of people from other areas. And maybe I wouldn't have the opportunity to have it, if it wasn't for that space. So I could hear how the nursing student feels in our relationships, with patients, as professionals. And all this made us review, even when we put ourselves in the position of a patient and remember some care that was good, some care that was not. And so, all this was very valid, right.” (FG-TA, F, Med)

Repercussions in relation to self, others, and professional life

Participants reported that the experience was transformative. It produced lasting knowledge, with repercussions on professional identity, defining the way of being a doctor. They also stated that it is present in daily practice, as a watershed moment from an academic, professional, communicational, and professional point of view:

“... that it’s a class, right, with all the methodologies applied, it makes us think outside the box, right, it’s a class that encourages us to develop different skills, so, bringing a little bit, right, of the staging and the development of the whole artistic part, right, you are invited to act scenes out, to be a scriptwriter, right, of a story, to write that story, to be a cameraperson also in some situations, right, to direct scenes.” (FG-HP, M, Med)

They also commented on experiences that consolidated professional identity and that the distinguished perspective on people will be part of professional and non-professional life, such as “saying ‘good morning’ to the doorperson”. Both the construction and the reconstruction of being through experience in the subject were highlighted:

“… a reconstruction guided by the thinking of the class.” (FG-HP, M, Med)

“So, there was information, there were experiences that helped me to outline the type of professional I want to be, right, which is about always having this positioning of listening, of trying to understand the other person’s side and the class certainly contributed to this.” (FG-TA, F, Med)

They stated that experience transforms the life of doctors, that of other professionals who take the subject, and that of the people who will receive care from these professionals:

“… we need to think about the kind of world we want to have, right, and if change starts with us, we have to stop to reflect on how we are acting as human beings to also act as health professionals, and I remember the class very well.” (FG-S, F, Pha)

“We change a lot when we realize that we have this power to transform a very awkward situation into a situation that is not so bad, and this only happens through the communication process, without a doubt.” (FG-HP, M, Med)

Experience in the subject reverberated in a culture of peace due to assertivity that makes it possible to deal with strong emotions. It reverberated in interprofessional work “without dispute over knowledge”, with reduced rivalries and the recognition of professions as a complement between areas of health, promoting recognition of others’ work and applying the lived experience from the discipline to their lives years later. Participants emphasized that the experience in the CH subject made it possible to understand others and themselves:

“What I think was a big take-home point for my life is the question of understanding human beings in a complex way, you know? Understanding the person as a unit of a story, a person who has gone through a specific story, who went through experiences that belong to him/her, and there are several feelings that permeate him/her, which I don’t know and I need to respect him/her with this in mind. And have a minimum of healthy communication to be able to guide them in the face of important information for their treatment, or to communicate bad news. And that’s what I think I’m going to carry with me in my life, I think, a different perspective of the person, if I could sum it up, you know?” (FG-TA, M, Med)

Participants also reported that this meaningful and inspiring experience is applicable to professional practice. In relation to the experience’s repercussions on academic life, participants stated that, by promoting mental health, relaxation, and well-being and reducing anxiety, “it recharges energy”, initiates students into teaching and research, and improves teamwork. There was also an impact on living together, promoting valuing of the individual, the search for the other, respect, and sensitivity, also between colleagues from different areas.

With respect to the repercussions on professional life, “it makes a glaring difference” in medical practice, which reverberates in the improvement of the health service and in interprofessional life.

### Reflections about the curriculum, interprofessional dialogue, and formation

The interdisciplinarity, empathy, and hope for better interpersonal relationships were part of the essence of the class, which was maintained throughout the timeline, as it went through different phases and semesters. The multidisciplinary format of the class and conversation circles encouraged interprofessional dialogue in health education. Regarding professional formation, participants reflected that the CH subject promotes humanization:

“The day-to-day routine is more aggressive, the routine is hard, and if you lose that feeling, this time […] of Communication in Healthcare, it can harm the rest of your professional life, right, because the tendency is for you to harden yourself as a professional […] to set a little bit aside, and this class is really needed. It is the humanization of the profession.” (FG-TA, M, Med)

One of the reflections about CH was that the class inserts active methods, ludicity, and art in the context of health courses that have a traditional curricular matrix:

“… a paradigm shift in the lives of students who take it, seeing that the Medicine course is an extremely Cartesian course, and all of a sudden you have a proposal with a more holistic approach, […] and this paradigm shift, at the same time that it startles us, leads to understanding new environments.” (FG-HP, M, Med)

Another recurrent reflection in all FGs was that it should be a mandatory part of the curriculum, and everyone should have this experience:

“… the class […] really enriches the people who are participating, it enriches the people who are watching, the person who is teaching... It is a class that there should be, the way you teach it, at all universities, it should be a pilot project for other universities that don’t have the class yet or anything similar or anything aimed at humanizing the health area.” (FG-S, M, Med)

On this topic, it was also reported that, even though there were so many changes over time, there exists an essence, a value that is maintained in the curriculum of CH:

“… we’re saying the same thing, but each one with their own particularities, but, like, the question of it being a safe environment, an environment where we have speech, have a voice, have autonomy, in short, we are saying the same thing, and we lived the class at very different times, in different years, and that’s very nice.” (FG-TA, F, Med)

Furthermore, participants stated that communication per se signifies a means of promoting health:

“So, communicating is promoting health, it is important for us to be able to communicate, for us to send an edifying message, to send a clear message to patients and obviously to colleagues, in short, to the team.” (FG-HP, M, Med)

## Discussion

In summary, the participants reported that the teaching-learning experience in CH was important, and it contributed to the formation of professional identity, improving interpersonal relationships and interprofessionalism, with repercussions on patient care. The main motivation for signing up was encouragement from friends who had taken the subject. Prior expectations about the course involved learning to welcome patients and having contact with the universe of art. There was a positive break in expectations for those who were expecting more of the same, or be it, a methodology following the traditional model, but they were faced with active methods, mediated by art. Meanings of the experience included interdisciplinarity, empathy, and hope for better interpersonal relationships. The shaping moments were related to understanding the other beyond the health complaint and integrative dynamics, such as the “care corridor” and artistic presentations, as well as moments in the student-teacher relationship. The teaching-learning experience was retained in a defining manner through the teaching method, with creative contributions in the ludic-artistic-reflective seminars. Regarding the types of experiences retained, there was the practice of empathy in daily communication, in the way of breaking bad news, and in attitudes towards multidisciplinary work. Regarding the repercussions in relation to self, others, and professional career, participants stated that the experience was transformative, producing lasting knowledge and defining professional identity. They also highlighted that the repercussions are present in daily practice. In relation to the reflections about the curriculum, interprofessional dialogue, and formation, participants indicated that there is an essence of the class that was maintained throughout the timeline, going through different phases and different semesters. They said that interprofessional dialogue is encouraged within health education, because of the multidisciplinary format of the discipline. They also highlighted that the class should be mandatory, as it represented the humanization of health professions.

From this point of view, there were repercussions on professional identity, for example, the way of being and becoming a doctor. They reported that the experience had repercussions on the formation of being, which are always present in professional practice, relationship with patients, with other professionals in the team. And they concluded that the learning acquired at that time can be applied today. Participants also reflected that this research with FGs contributed to reliving the experience, recovering defining moments and lived moments in the class with more present awareness. Participants attribute the message communicated in the class as one that breaks the paradigm in medical education.^46^ This is in agreement with a study that found that the moments that generate profound feelings of awareness in students are often moments that would not be recognizable (even post hoc) as remarkable by others.^47^

There is a set of values, known as the hidden curriculum, which is passed on between the lines by positive or negative attitudes. A positive example cited was the teacher arriving on a bicycle to teach classes, which speaks volumes more than words regarding environmental awareness, as indicated in the Results. Accordingly, experiences of personal and professional development and consolidation of professional identity were narrated, characterizing what Gadamer refers to as “Bildung,” namely, the construction of self, with respect to formation and development of “being-in-the-world”.^22^^,^^23^

Another important point of reflection occurred in the student-teacher relationship, which configures a terrain of safety and openness to dialogue. In this case, the student-teacher relationship and the class experience contributed to open and authentic dialogue, consolidating the teaching-learning proposal. This relationship also permeates the hidden curriculum and the formation of professional identity, impacting the mental health of students. The autonomy given to students was another value passed on within this relationship contributing to students’ mental health.^48^

Other topic discussed by all groups was interprofessionalism. The importance of bringing together students from different health courses in a single class emerged, and this practice was reported to broaden the vision and appreciation of diverse health professions, increasing mutual respect, reducing dispute over knowledge, and influencing the formation of professional identity, which was another recurring topic in all groups. Another innovation that emerged from the FGs is related to the multiple aspects of the class; these strategies have promoted experiences of interaction, interprofessionalism, and respect, with a positive impact on patient care. Other studies have indicated that an interprofessional model contributed to improving the performance of the team and promoting health, improving the quality of the work process and patient care.^49^^,^^50^ This study on the experience in CH is in accordance with the Best Evidence Medical Education (BEME) systematic review, which, based on the modified Kirkpatrick model, found “that learners respond well to interprofessional education, their attitudes and perceptions of one another improve, and they report increases in collaborative knowledge and skills,” with evidence of benefits to patients.^51^

Motivations and expectations in signing up for CH involved contact with the world of art and the humanization of the profession. Active, student-centered, and art-mediated methods make it possible to retain the teaching-learning experience in a defining manner, stimulating creativity through ludic-artistic-reflective seminars with dramatization, music, dance, video scripting, poetry, and relaxed dialogues. The teaching-learning strategies and the method of the CH subject systematize, without making the teaching-learning process rigid, guaranteeing a creative and welcoming space. These strategies, including the steps for ludic-artistic-reflective seminars created in the class, produce a series of new experiences that are never repeated, where something new always comes. These seminars, which were conducted by students under the supervision of TAs and educators, enrich both those who attend and those who elaborate the themes for the class meeting, making the experience transforming. A systematic review and meta-analysis selected 49 empirical studies and conceptual articles about the use of creative arts, imagery and symbolism in the context of professional education. The authors concluded, at each of these steps, specific actions by the teacher can enhance the potential for learners to move to the next step. The process can be enhanced when learners participate in the context of a group, and the group itself can undergo transformative change.^52^ Furthermore, in relation to art, play, and celebration, which are Gadamerian concepts that express the way of “being-in-the-world” and interacting with one other, bringing art as a strong channel for understanding life, we can relate participants’ artistic expressions and interactions in the integrative dynamics.^53^

Still in relation to the method of the discipline with ludic-artistic-reflexive seminars and to the importance of the student-teacher relationship in the educational processes, the term “didactic choreography” is present in the literature, where it refers to the teacher-student relationship in the educational process, with previously defined teaching-learning steps.^54^ The method developed in CH with 10 steps for ludic-artistic-reflective seminars provides stability and predictability, serving as an axis for class meetings, reducing anxiety and the terrain of the unexpected, as stated in the Results. Expression with art and ludicity represented unwinding, relaxation, reflection on care for patients and self, a moment of self-revelation to others, and an open space for subjectivity within the environment of university education, which Gadamer refers to as openness to understanding the world.^55^^,^^56^ Students’ productions in integrative dynamics, such as the corridor of care, were identified as one of the most eagerly awaited moments of class meetings and those that promoted greater integration through art, festival, and ludicity. In relation to ludicity, Gadamer compares festivals and games to the field where relationships take place and where people may experience what is lived with their own style and personal identity. These strategies represent a hermeneutic exercise, comprehension of others, empathy, a game of understanding, and the production of care.^53^ Dramatizations, for example, “Theater of the Oppressed” and videos contextualizing real-life situations, prepare participants for what will take place during patient care, as preliminary rehearsal for professional practice.^57^ In this sense, dialogue and artistic expression also help capture experience, contributing to professional formation and research in medical education. For example, another study in the Brazilian context used art in the form of rich picture drawings, to capture the experience of medical students in the face of moral dilemmas during formation, indicating that it is a difficult experience and students need more spaces for dialogue, care, and self-care during their education.^58^ In order to respond to these attitudinal aspects, CH, by using art and ludicity as a strategy, promotes reflections on the dilemmas of health professions and promotes well-being in the academic environment.

According to FG participants, the CH class is an educational intervention with diverse innovations within the context in which it is inserted. These include the use of active methods in a medical course with a conventional curriculum, as well as a student-centered approach, which is in agreement with research on the adaptive curriculum.^59^

All FGs suggested that CH become a mandatory class in the medical course, as well as the pilot project implemented with the same methodology in other universities, as the approach represents the humanization of health professions. Accordingly, it is worth highlighting that, in another context, a study carried out in the state of New York, on an elective interprofessional subject that also works with empathy and communication for students of Medicine, Nursing, and Dentistry resulted in the incorporation of this educational activity in the pre-clinical curriculum of Medicine, maintaining it as an elective subject for other health courses.^60^

The moments lived in the class have shown applications in practice, and they were consolidated in the FGs. This re-elaboration of the experience represents an unexpected result that went beyond the research objectives, contributing to the idea of isolation for the sake of health and social solidarity during the pandemic.^61^

In October 2022, the Brazilian Association of Medical Education published the consensus for teaching communication in Brazilian medical schools, guiding the minimum contents to be taught, for the first time in Brazil.^62^ The importance of this consensus lies in the fact that Brazil’s National Curricular Guidelines for Undergraduate Courses in Healthcare state that the teaching of communication is important in health; however, they do not specify what should be taught or how it should be taught. The third author of the present qualitative study coordinated the construction of the consensus. The first author was involved in the process of developing it. It is worth emphasizing that CH, even though it was initiated before the publication of the consensus, is in agreement with its contents. Furthermore, CH contributes by indicating a teaching-learning methodology for health educators to facilitate these contents in the classroom health; in other words, CH encompasses both the what and the how, with respect to teaching-learning communication in health. From the point of view of education and research, this qualitative evaluation of this intervention CH contributes by investigating the teaching-learning experience, opening paths in the practices of educators and researchers in Brazil and worldwide.

In relation to our study limitations, our findings relate to the specific context in a semi-arid region in Brazil, in undergraduate courses for health professional, with characteristics that are singular and may thus be limited. Nevertheless, this is the first study in this region that seeks to understand relational experiences in health education based on a multiprofessional elective class, which develops health communication skills in a way that is innovative in the context where it is inserted.

Methodologically, the approach that associates Philosophical Hermeneutics with thematic content analysis is still uncommon, even though there is a study from the same macroregion of Brazil; on the other hand, it contributes to consolidating new ways of understanding the experience.

The fact that the first author and moderator of the focus group also was a professor of the course is important due to the hermeneutic value that made possible the fusion of horizons of perspectives. Accordingly, by knowing and being part of the experience, it was possible to formulate relevant questions and deepen the pre-elaborated guiding questions for the interview. However, this fact could, to some extent, affect the participants’ answers. The pilot group, which calibrated the interview guide and the online platform, valued the participation of internal and external mediators to CH. This is similar to a study from Denmark that sought to understand doctors’ experience in a mandatory postgraduate course on communication skills for residents in internal medicine, general medicine and oncology. The study also applied focus groups, mediated by researchers, including a professor of the discipline.^63^

## Conclusions

In relation to the contributions to professional formation and medical education, the teaching-learning experience of participants in the CH class contributed to improvement of “being” in the personal and professional sense of those involved in the experience. It signified a path for expression, subjectivity, understanding, living together, dialogue, autonomy, respect, art, reflection, ludicity, and well-being, elevating care for patients during professional practice. The 10 steps for ludic-artistic-reflective seminars developed in the environment of the CH class can be applied to create novel and publishable experience reports, contributing to interprofessional dialogue and to the art of healing. The teaching-learning experience lived in the CH class promoted transformations in participants’ ways of being, knowing, and acting, producing meanings that go beyond the teaching-learning, cognitive-conceptual relation. Thus, the didactic steps of the ludic-artistic-reflective seminars, created in the environment of the CH class, provided a truly formative experience, accessing and reaching the being of participants by means of art and play, promoting forms of communication with a view of solidarity, enhancing the process of living together in interprofessional teams and bringing people closer together in understanding the art of caring.

Accordingly, the research results bring implications for medical education with respect to paths for teaching-learning of communication skills, empathy, genuine dialogue, and interprofessionalism. The experience in CH indicates a teaching-learning methodology that, in addition to covering contents specified in the Brazilian consensus, opens a promising path as to how to develop them in the classroom of national and international educators and researchers. Finally, this study contributes to medical education by bringing the point of view of students and health professionals who took CH and how they apply the teaching-learning in professional practice. Therefore, we recommend further studies with a philosophical hermeneutic framework and online focus groups, which reduce distances, bring people together, and make it possible to deepen the understanding of educational interventions in the area of medical education, as well as in health care and professional formation.

### Acknowledgments

The authors would like to thank Robert Bradley Smith of 4 Sílabas for his translation, research assistance, and editing services throughout the study; Prof. Dr. Ludovic Aubin for his teachings about assertive communication; Prof. Bemmerval Augusto Nogueira Gomes for transcribing the focus group interviews; Prof. Dr. Patricia Gomes de Matos Bezerra for initial advisorship of the research project (2017 to 2018), suggesting the research object focus on the Communication in Healthcare elective course; Prof. Dr. Marcelo Ribeiro for co-advisorship (2019 to 2020), for bringing the theoretical-philosophical framework, and for mediating the pilot, teaching assistant, and student focus groups; the members of the doctoral defense committee, Prof. Dr. Lygia Carmen Vanderlei, Prof. Dr. Malaquias Batista Filho, Prof. Dr. Gabriel Kafure da Rocha, Prof. Dr. Juliana de Farias Pessoa Guerra, and Prof. Dr. Tereza Rebecca de Melo e Lima for their contributions; the Foundation for Advancement of International Medical Education and Research (FAIMER) for their teachings about research and medical education. The authors would like to thank Prof. Dr. Shirley Macedo and Prof. Dr. Darlindo Ferreira de Lima of the Laboratory of Transdisciplinary Practices in Health and Education (Laboratório de Práticas Transdisciplinares em Saúde e Educação, Universidade Federal do Vale do São Francisco - LETRANS/UNIVASF) for their support and collaboration and for clarifying the research object with a focus on understanding the lived experience, which contemplates and goes beyond studies that investigate perceptions; Prof. Dr. Carlos Eduardo Menezes Amaral of the Department of Medicine, UNIVASF, Paulo Afonso Campus for his support; Prof. Dr. José Eulálio Cabral Filho and Prof. Dr. Suely Arruda Vidal of the IMIP Postgraduate Program for their support and teaching; Prof. Dr. Gustavo Silvano Batista and Prof. Dr. Fábio Solon Tajra of the Postgraduate Program of the Federal University of Piauí (UFPI), for their teachings about hermeneutic philosophy; Bernadete Zimmerle for initial contributions to data analysis; the teaching assistant Mariana Pereira and the student Cristiane Almeida for their support; and all the students who participated with their lived educational and professional experiences.

### Conflict of Interest

The authors declare that they have no conflict of interest.
